# Shaping Soft Robotic Microactuators by Wire Electrical Discharge Grinding

**DOI:** 10.3390/mi11070661

**Published:** 2020-07-04

**Authors:** Edoardo Milana, Mattia Bellotti, Benjamin Gorissen, Jun Qian, Michaël De Volder, Dominiek Reynaerts

**Affiliations:** 1 Department of Mechanical Engineering, KU Leuven and Flanders Make, Celestijnenlaan 300, 3001 Leuven, Belgium; edoardo.milana@kuleuven.be (E.M.); mattia.bellotti@kuleuven.be (M.B.); benjamin.gorissen@kuleuven.be (B.G.); jun.qian@kuleuven.be (J.Q.); mfld2@cam.ac.uk (M.D.V.); 2 Harvard John A. Paulson School of Engineering and Applied Sciences, Harvard University, Cambridge, MA 02138, USA; 3 Institute for Manufacturing, Department of Engineering, University of Cambridge, 17 Charles Babbage Road, Cambridge CB3 0FS, UK

**Keywords:** wire electrical discharge grinding (WEDG), micromoulding, soft microrobotics, electrical discharge machining (EDM)

## Abstract

Inflatable soft microactuators typically consist of an elastic material with an internal void that can be inflated to generate a deformation. A crucial feature of these actuators is the shape of ther inflatable void as it determines the bending motion. Due to fabrication limitations, low complex void geometries are the de facto standard, severely restricting attainable motions. This paper introduces wire electrical discharge grinding (WEDG) for shaping the inflatable void, increasing their complexity. This approach enables the creation of new deformation patterns and functionalities. The WEDG process is used to create various moulds to cast rubber microactuators. These microactuators are fabricated through a bonding-free micromoulding process, which is highly sensitive to the accuracy of the mould. The mould cavity (outside of the actuator) is defined by micromilling, whereas the mould insert (inner cavity of the actuator) is defined by WEDG. The deformation patterns are evaluated with a multi-segment linear bending model. The produced microactuators are also characterised and compared with respect to the morphology of the inner cavity. All microactuators have a cylindrical shape with a length of 8 mm and a diameter of 0.8 mm. Actuation tests at a maximum pressure of 50 kPa indicate that complex deformation patterns such as curling, differential bending or multi-points bending can be achieved.

## 1. Introduction

Soft robotic systems are capturing the interests of scientists and engineers with characteristics that are breaking with conventional robot traditions. Softness, compliancy and cost-effective manufacturing make soft robots preferable in applications where gentle manipulation and human interaction occur [1]. Thanks to their low mechanical stiffness, soft robots can safely operate in unstructured environments by adapting to unforeseen collisions and reduce the risk of harmful events [2]. For those reasons, soft robotic technology is extensively utilised to make universal grippers [3], some of which have been commercialised [4]. Other promising soft robotic applications include robots for search and rescue operations [5,6] as well as innovative techniques to make untethered and entirely soft machines [7]. There is an increasing interest in downscaling soft robotic technology to micrometre scales to drive advances in applications where the operational environment is unpredictable or extremely delicate, such as minimally invasive surgery and drug delivery [8,9,10]. Further, soft microrobotics has already been applied in microfluidics for making flexible active valves [11] and artificial cilia for biomimetic micromixing and micropumping [12].

Soft robots necessitate actuators that display large deformations as a response to a generalised force input. Typically, soft actuators are designed such that their deformation corresponds to a desired kinematic trajectory, behaving as a compliant mechanism with one degree of freedom. Many types of soft actuators can be distinguished according to the nature of the applied forces [8], which can vary from electrical (dielectric or ionic polymers) to magnetic fields (magnetic polymers), from solvent concentrations (hydrogels) to pressurised fluids (inflatable structures), etc.

Elastic inflatable actuators (EIAs) are one of the most widespread soft actuators, that rely for their motions on the morphology of the actuators [13]. Such actuators at small-scale were first introduced by Suzumori [14,15] and Konishi [9,16] for biomedical applications. For a comprehensive review of the different types of EIAs we referred to previous studies (see [13,17]). Current miniaturised EIAs are made with a single inflatable elastomeric cavity which leads to a simple motion that can be bending [18], twisting [19], contracting [20] or extending [21]. Large-scale actuators, on the other hand, have been presented with richer deformations, originating from a more complex design. However, these complex designs are challenging to copy at smaller scales due to manufacturing limitations. At a larger scale, inflatable actuators are typically made out of different elastomeric parts, which are subsequently bonded or glued together. This approach makes it possible to create intricate actuator geometries that drastically modify the actuator performance as shown by Mosadegh et al. [22] for pneumatic networks (PneuNets) bending actuators. However, at smaller scales, creating similar actuators by combining parts and bonding them is an uphill task, due to more stringent requirements on the tolerances of the parts to be assembled, possible misalignments and handling problems. A commonly used bonding process for sub-centimetre soft actuators in polydimethylsiloxane (PDMS) consists of oxygen plasma treatment to activate the PDMS surfaces before bonding [23]. However, these bonds are often the weakest part of the structure which eventually causes the actuator to rupture.

In order to circumvent these issues, we propose a bonding-free technique to fabricate millimetre-scaled soft bending microactuators, using out-of-plane moulding [18]. These microactuators consist of PDMS cylindrical structures with a simple cylindrical inflatable cavity, which is placed eccentric to the axis of the outer cylinder of the actuator. This eccentricity introduces an asymmetry in the cross-section of the actuator that causes the actuator to bend. The mould is composed of two micro-milled parts and a cylindrical microrod placed in between the two parts, where the shape of the microrod is replicated as the inflatable cavity. We used these devices for different applications such as artificial cilia [24] and flexible endoscopes [25]. However, given the simple morphology of the inflatable cavity, these microactuators have a limited operating range with a maximum bending angle of up to 45° [25].

In this paper we introduce an additional manufacturing step to this bonding-free technique in order to fabricate more complex microactuators. During this additional step, the cylindrical microrods are machined using a wire electrical discharge grinding (WEDG) process. WEDG is a manufacturing process in which material is removed from a rotating tool using a running wire through high-frequency sequences of electric discharges. WEDG, which was early developed by Masuzawa et al. [26], has been established over the past years as a proven technology for machining axisymmetric microrods down to less than 10 µm in diameter [27]. Nowadays, WEDG is used for machining not only cylindrical, but also tapered microrods [28] as well as microrods with a spherical tip [29]. WEDG’ed microrods find a wide variety of applications in industry such as touch probes for contact measurement systems [30] or microtools for drilling tapered microholes [31] and microhole arrays [32].

WEDG was already used for the fabrication of classic pneumatic microactuators [33], but not for soft actuators. Here, WEDG is used for machining axisymmetric microrods with more complex shapes to be placed in the internal cavity of inflatable soft robotic actuators in a micromoulding process. Dimensional and surface metrology are used to check that the accuracy and surface quality of the fabricated microrods are compatible with the moulding requirements. Further, an analytical model is used to predict the deformation of the moulded actuator. The model is validated by experimental tests on prototypes. These tests clearly show the benefits in terms of deformation patterns of using structured microrods over their unstructured counterparts. The paper is structured as follows: in the first section we report on three different morphologies of inflatable cavities and the details about the WEDG process to realise the respective internal shapes. Subsequently the manufacturing steps involved in the moulding process are described. Finally, the microactuators are characterised and compared to the state-of-the-art.

## 2. Materials and Methods

### 2.1. Inflatable Cavity Morphologies

**Figure 1:**
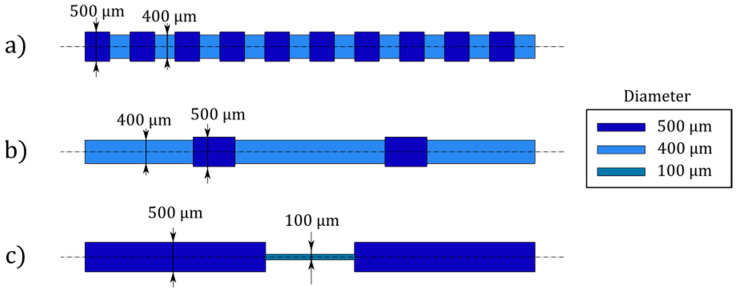
Inflatable cavity morphologies: (**a**) *Saw*, (**b**) *Totem*, (**c**) *Halter*. The cavities are axisymmetric, and the colour code corresponds to the local diameter of each segment. The segment lengths are reported in Table 1.

All microactuators described in this work have the same general architecture and only differ in the shape of the inflatable cavities. Nevertheless, the deformation changes significantly as further reported. The microactuators are cylindrical pillars, 8 mm in length and 0.8 mm in outer diameter, with inflatable cavities that are placed at an eccentricity, *e*, of 110 µm. We designed the inflatable cavities to be compatible with the WEDG process as described in Section 2.2. Thus, all cavities are axisymmetric with local variations of the radius across the length. We identified three different actuators with distinct rod shapes that lead to different functional deformations. In the paper we refer to them as: (i) *Saw* (Figure 1a); (ii) *Totem* (Figure 1b); (iii) *Halter* (Figure 1c).

The inflatable cavities of the three actuators can be divided into segments of different length and diameter. The segment lengths are reported in Table 1, while the diameters can be found in Figure 1, where the colour codes distinguish the different segments. In total, the combined length of the inflatable cavities equals 7.5 mm.

**Figure 2:**
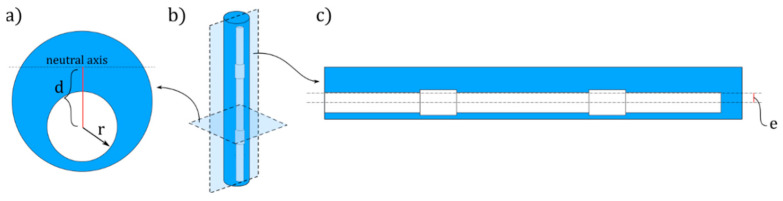
(**a**) Cross section of the microactuators. The distance between the neutral axis and the centre of the inflatable cavity (in white) is the moment arm *d*. (**b**) 3D representation of the microactuator with a *Totem* morphology of the inflatable chamber. Section planes are displayed. (**c**) Longitudinal section of the microactuator. The distance between the axis of the inflatable cavity and that of the rubber structure corresponds to the eccentricity, *e*.

Given the eccentricity of the cavity with respect to the axis of symmetry of the structure, all actuators undergo a bending motion upon pressurisation, as explained in our previous paper [18]. Due to the asymmetric placement of the central void (Figure 2a), the centroid of pressure is shifted with respect to the neutral (bending) axis of the structure, resulting in a large bending deformation. In Figure 2b,c, the 3D representation and longitudinal cross-section of a microactuator of *Totem* type are shown as an example. The eccentricity, *e*, corresponds to the distance between the axis of the inflatable cavity and that of the rubber structure.

### 2.2. WEDG Process

**Figure 3:**
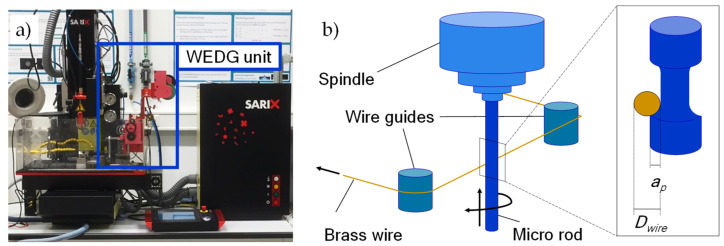
(**a**) Experimental setup and (**b**) schematic view of the wire electrical discharge grinding (WEDG) process. In the magnified view in (b), the wire diameter (*D*_wire_) and the depth of cut (*a*_p_) are indicated. The figure is adapted from [34].

WEDG is used for shaping the microrods to the desired shapes. In this research, we developed a WEDG processing strategy using the WEDG unit of a SARIX^®^ SX-100-HPM micro-EDM machine (Figure 3a). This unit is equipped with a brass wire of 200 μm in diameter (*D_wire_*), which is used for machining the microrods. The brass wire runs continuously during the WEDG process. Cylindrical microrods in tungsten carbide provided by SARIX^®^ are used. The microrods, which have a nominal diameter (*D_rod_*) of 500 μm, are clamped in the spindle of the micro-EDM machine tool. In Figure 3b, a schematic illustration of the performed WEDG process is shown. During the WEDG process, hydrocarbon oil of viscosity equal to 2.4 mm^2^/s at room temperature (HEDMA^®^ 111) is applied as a dielectric fluid.

In order to reduce machining time, WEDG processing is carried out using two machining regimes: roughing and finishing. Table 2 lists the processing parameters applied in each machining regime. In both cases, a positive polarity is applied to the microrod. In roughing, a relatively high amount of energy per discharge (average discharge energy: 46.5 µJ) is applied in order to increase the machining efficiency. The amount of energy per discharge is reduced during finishing (average discharge energy: 3.8 µJ). The average energy per discharge is computed from samples of the voltage and current signals including roughly 2000 pulses following the same methodologies we applied in a previous study [35].

The WEDG process for shaping the microrods consists of multiple roughing steps and a single finishing step. A radial depth of cut *(a_p_*) equal to 20 µm is applied during the roughing step. This value is chosen based on the results of some preliminary experiments, which were carried out to maximise the material removal rate (MRR) during roughing [34]. In the finishing step, a 10 µm radial depth of cut is applied. The finishing step is meant to ensure a higher machining accuracy and improve the surface quality of the microrods. In order to assess the accuracy and quality of the WEDG’ed sections on the microrods, post-process metrology is carried out by optical microscopy (ZEISS^®^ SteREO Discovery V20), confocal microscopy (Sensofar^®^ S lynx) and scanning electron microscopy (Phenom^®^ Pro).

### 2.3. Moulding

**Figure 4:**
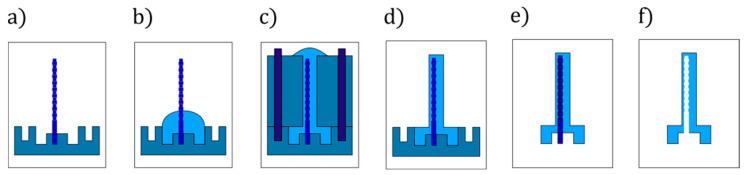
Fabrication process steps. (**a**) WEDG’ed (wire electrical discharge grinding) microrod is placed in the designated hole of the aluminium bottom half of the mould. (**b**) Uncured rubber is poured on the bottom half. (**c**) The top half is aligned and tightened to the bottom half. (**d**) After curing, the top part is removed. (**e**) Microactuator demoulding. (**f**) Microrod removal (figure adapted from [36]).

The microactuators are fabricated through a bonding-free out-of-plane moulding process, as sketched in Figure 4. The mould consists of two aluminium micromilled parts. The bottom part contains the designated holes for the microrods (Figure 4a) as well as features for releasing the microactuators, while the top part presents through-holes with a diameter equal to the external diameter of the microactuators (0.8 mm). The holes in the two parts are drilled in such positions so that when aligned and assembled they create an eccentricity of 110 µm of the inflatable cavity. Before pouring the uncured rubber, the mould surfaces are coated with a layer of release agent (Devcon).

The two liquid prepolymers of the silicone rubber (Dragon Skin™ 30 by Smooth-On) are thoroughly mixed in a 1:1 ratio for 2 min. The uncured rubber is subsequently placed in a vacuum chamber for 5 min to make sure no air is trapped inside. Indeed, this is a fundamental step as the presence of microscopic air bubbles in the cured rubber dramatically affects the mechanical properties, and due to the small size of the actuators also cause imperfections and unwanted voids.

After filling the bottom part of the mould (Figure 4b), we degas it again for 5 min in the vacuum chamber. The top part of the mould is aligned with respect to the bottom part through alignment pins and firmly tightened (Figure 4c). This tightening ensures that the uncured rubber flows in all the features of the mould. The mould is subsequently placed in the oven at 60 °C for 1 h to let the elastomer cure. After curing, the mould is opened, using ethanol as lubricant (Figure 4d). Given their intricate shape, the microrods are stuck in the microactuators after demoulding (Figure 4e). Removing the microrods is the last and most delicate step, which requires the use of ethanol to slightly swell the silicone and allow a safe removal of the rod (Figure 4f). Since the rubber cures around the microrod, the absence of air generates a negative relative pressure that locks the microrod inside the microactuator. The function of ethanol is to both lubricate and swell the rubber so that air can penetrate the cavity and eliminate the negative relative pressure. This effect combined with the compliancy of the soft material enables the removal of the microrod. After removing the microrod, the microactuators are dried at room temperature to fully evaporate the ethanol. Then, they can be connected to pressure supply tubing.

Microactuators of the *Halter* type require a different process to remove the microrod. Indeed, due to the large diameter variation (from 500 µm to 100 µm), the swelling-induced removal of the microrod is not effective. Alternatively, the microrod is broken at the thin section and the two parts are extracted from the base and the tip of the microactuator. The tip is subsequently sealed with a drop of uncured rubber.

### 2.4. Analytical Model

In order to evaluate the deformation patterns associated with the different inflatable cavity morphologies of the actuators, we apply a multi-segment linear analytical model consisting of Euler–Bernoulli beam segments that are each loaded with a constant bending moment. For one segment, this model has already been applied to capture the overall bending deformation of a soft bender [18,37,38], while here we introduce a segmented approach. The different actuator types that we analyse in this work can be divided into *n* segments (Table 1), where each radial variation *r_i_* is considered as the *i*-th segment (*i =* 1,…, *n*) of length *l_i_*. In our model, each segment is subjected to a constant moment *M_i_*

(1) $$M_{i}=p_{E}\pi r_{i}^{2}d_{i},$$

where *d_i_* is the distance between the centre of the cavity section and the neutral bending axis, passing through the centre of mass of the section (Figure 2 and Equation (2)), while *p_E_* is a non-dimensional parameter corresponding to the normalised pressure with respect to the Young’s modulus of the material ($p_{E}=p/E)$.

(2) $$d_{i}=e+\frac{er_{i}^{2}}{R^{2}-r_{i}^{2}}$$

Therefore, the curvature of the *i*-th segment is equal to

(3) $$k_{i}=\frac{M_{i}}{I_{i}},$$

where *I_i_* is the second moment of area of the section:

(4) $$I_{i}=\frac{\pi \left(R^{4}-r_{i}^{4}\right)}{4}+\pi R^{2}{\left(d_{i}-e\right)}^{2}-\pi r_{i}^{2}d_{i}^{2}$$

The curved profiles of the *n* segments are numerically computed and assembled in MATLAB to show the overall deformed configuration of the actuator.

### 2.5. Microactuators Experimental Setup

The experimental setup to characterise the microactuator response consists of a pressure regulator valve (Festo LR-D-7-I-Mini) fed with compressed air coupled to a manometer (Festo FMAP-63-1-1/4-EN) and connected to the tested microactuator. A 500 µm outer diameter (OD) tube is inserted in the inflatable cavity of the microactuator and fixed with uncured silicone rubber. In the experiment, the pressure input is manually increased by 10 kPa increments, while a camera (Nikon 1 V3) captures the actuator deformation in the bending plane once a static equilibrium is reached at each pressure increment. As the actuators have different curvatures in accordance to their segmentation, the deformed configurations are characterised using the tip trajectory as parameter.

## 3. Results and Discussions

### 3.1. WEDG Accuracy and Quality

**Figure 5:**
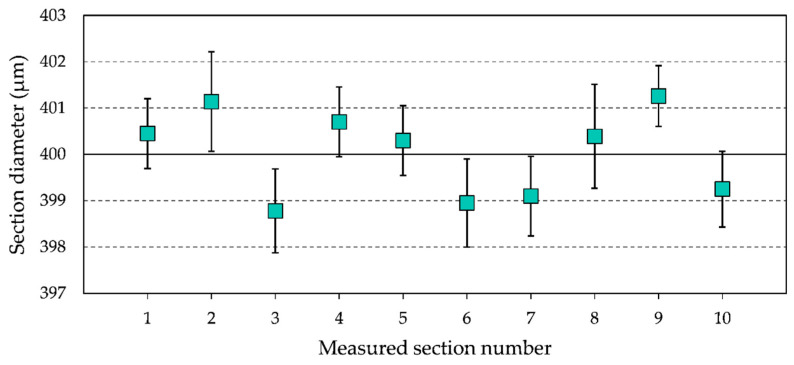
Measurement of the diameter of the WEDG’ed sections of the microrods. Ten different sections are measured, and eight measurements are carried out per section. The data points refer to the mean diameter of each measured section, while the error bars indicate the standard deviation of the eight measurements.

In order to evaluate the accuracy of the WEDG process for the fabrication of soft robotic microactuators, the WEDG’ed sections of the microrods are measured by means of a ZEISS^®^ SteREO Discovery V20 microscope. The measurement results for ten different sections, which were machined on microrods with a *Saw* morphology, are shown in Figure 5. The target diameter for the measured sections is 400 µm.

From Figure 5, it can be observed that the WEDG’ed sections deviate less than ±3 µm from the target diameter. In particular, the average diameter of the measured sections is 400.4 µm, while the standard deviation is 2.59 µm. These results highlight the high precision and accuracy of the proposed WEDG processing method. In order to study the effect of the processing accuracy on the performance of soft robotic microactuators, the analytical model presented in Section 2.4 is used to analyse the influence of variations to the diameter of the inflatable cavity of a segment on the curvature relative error (CRE). Equation (5) is used to compute the CRE from the curvature *k*_0_ of a segment of nominal diameter and the curvature *k*_e_ of a segment of diameter affected by a machining error.

(5) $$\mathrm{CRE}=\frac{\left|k_{e}-k_{0}\right|}{k_{0}}$$

**Figure 6:**
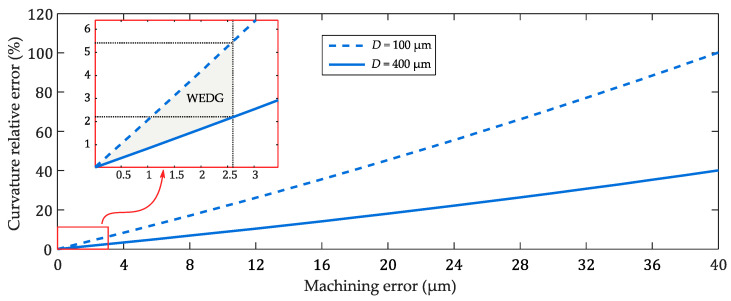
Effect of the machining error on the curvature relative error (CRE). In the graph, the calculated CRE of segments with inner cavity diameter *D* equal to 100 µm and 400 µm is shown.

In Figure 6, the effect of the machining error on the CRE is shown. Segments of diameter equal to 100 µm and 400 µm are considered, which are the diameters of the segments of the three types of microactuators presented in this work. It can be seen that the CRE linearly increases with the machining error. WEDG processing, which allows the machining of segments having a deviation of less than ±3 µm from the nominal diameter, results in maximum CREs of about 2.2% and 5.3% for segments of diameter equal to respectively 400 µm and 100 µm. These maximum errors are relatively small and confirm that WEDG processing can be considered as a viable technique for machining axisymmetric microrods to be used in the bonding-free fabrication process of soft robotic microactuators. The trends shown in Figure 6 also suggest that the finishing step is crucial and unavoidable for ensuring high performance of the soft robotic microactuators since deviations from the nominal diameter in the order of 4–10 µm are observed after roughing. These deviations would result in an increase of the CRE up to 20% for a section diameter of 100 µm.

The main drawback of the proposed WEDG method is the relatively long processing time. For instance, it takes approximately 5 min for machining a section of 400 µm diameter and 0.4 mm length on a cylindrical microrod of 500 µm diameter. In this case, the two roughing steps take approximately 3 min in total, while roughly 40% of the total machining time is spent in finishing. However, long processing time are acceptable, taking into consideration that a microrod can be used for moulding multiple soft robotic microactuators. A possible solution for reducing the WEDG processing time could be to increase the discharge energy during the roughing steps. This can be accomplished, for example, by increasing the capacitance or open voltage parameters [39]. Nevertheless, an increase of the discharge energy is likely to result in a more aggressive and less repeatable removal of material by electric discharges, thus decreasing the overall accuracy and precision of the WEDG process.

**Figure 7:**
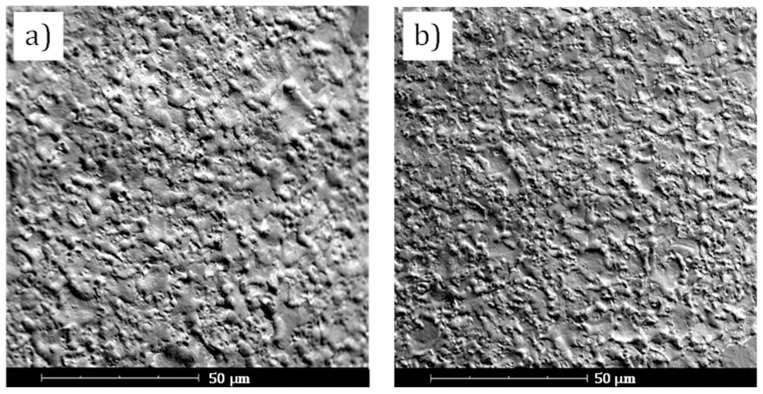
Surface morphology of a microrod (**a**) before and (**b**) after the finishing step. The images are taken by means of Phenom^®^ Pro scanning electron microscope.

The surface roughness of the WEDG’ed sections of the microrods is analysed qualitatively by means of scanning electron microscopy (SEM) and confocal microscopy. In order to study the effects of finishing on the surface quality, the surface analysis is carried out on the same sections considered in Figure 5 and on other sections, which were machined by interrupting the WEDG process before performing the final finishing step. The benefits of the finishing step are clear when observing the SEM micrographs of the microrods before and after the finishing step (Figure 7). In particular, it can be seen that a less uneven surface morphology can be achieved once the single-step finishing is performed after roughing. The observed difference corresponds to a decrease of the surface roughness from *S_a_* = 0.84 μm to *S_a_* = 0.37 μm. These values refer to the average values of the *S_a_* surface parameter, which are computed from 20 samples measured on 10 different grooves by a Sensofar^®^ S lynx microscope in confocal mode (magnification: ×50, field of view: 350 × 260 µm). The *S_a_* parameter represents the arithmetical mean height of a surface. It is the extension of the *R_a_* parameter (arithmetical mean height of a line) to the surface. In light of the bonding-free fabrication process of the microactuators, a reduction in surface roughness is advantageous, since the demoulding forces in microreplication processes depend on friction [40]. Despite the relatively long machining time, it can be concluded that the finishing step is crucial not only to achieve the required processing accuracy, but also to facilitate the removal of the microrod after moulding.

**Figure 8:**
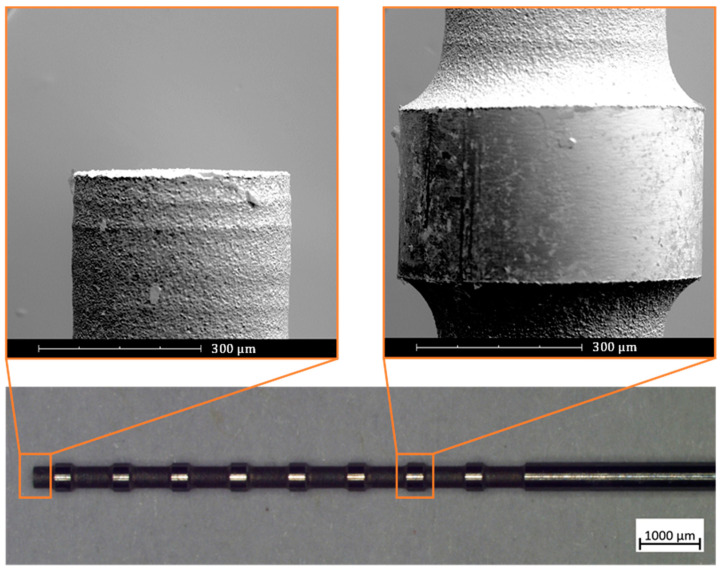
WEDG’ed microrod for moulding of soft pneumatic microactuators. The image of the microrod is taken using a ZEISS^®^ SteREO Discovery V20 optical microscope, while the enlarged views are taken by means of Phenom^®^ Pro scanning electron microscope.

Figure 8 shows a microrod after WEDG processing. The enlarged views taken by SEM reveal that a flat tip and straight edges can be achieved by WEDG. It can also be seen that the WEDG’ed sections have round chamfers, of which the radius depends on the radius of the wire which is used in the WEDG process. Round chamfers are crucial for demoulding the microactuators. Very sharp edges should indeed be avoided as they can damage the microactuators.

### 3.2. Microactuators Analytical Model

**Figure 9:**
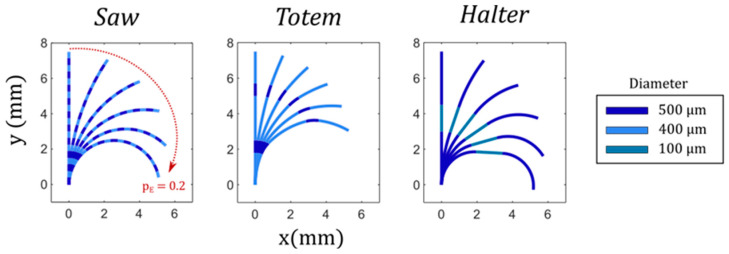
Analytical model results. Six configurations are depicted for each actuator at the same normalised pressure inputs (*p_E_*) varying from 0 (undeformed) to 0.2 (maximum deformation). Segments are distinguished with the same colour code as used in Figure 1.

The analytical model described in Section 2.4 is solved for the three different inflatable cavities. The normalised input pressure (*p_E_*) is equal for the three actuators, varying linearly from 0 to a maximum of 0.2. Figure 9 displays the deformation of the three actuators for six equidistant pressure values along the input ramp.

The different segments of each inflatable cavity are distinguished using the same colour code as for Figure 1. The sections of the inflatable cavity with a reduced diameter (light blue) undergo a lower curvature for two reasons. First of all, the bending stiffness is higher due to the increase of the second moment of area as the cross-sectional void (Figure 2) is smaller. Secondly, the normal force driving the bending moment scales linear with this cross-sectional area (Equation (1)).

The diverse responses of the segments along the microactuators determine the complex deformation patterns that we aim to achieve. As such, each low-stiffness segment acts as a compliant joint. For example, we expect *Saw* to achieve a full-curled configuration due to the higher distribution of joints, whereas *Totem* has a discrete deformation, with only two low-stiffness segments working as the main bending points. On the other hand, *Halter* bends only at the extremities of the microactuators while the central part stays undeformed.

### 3.3. Microactuators’ Characterisation

**Figure 10:**
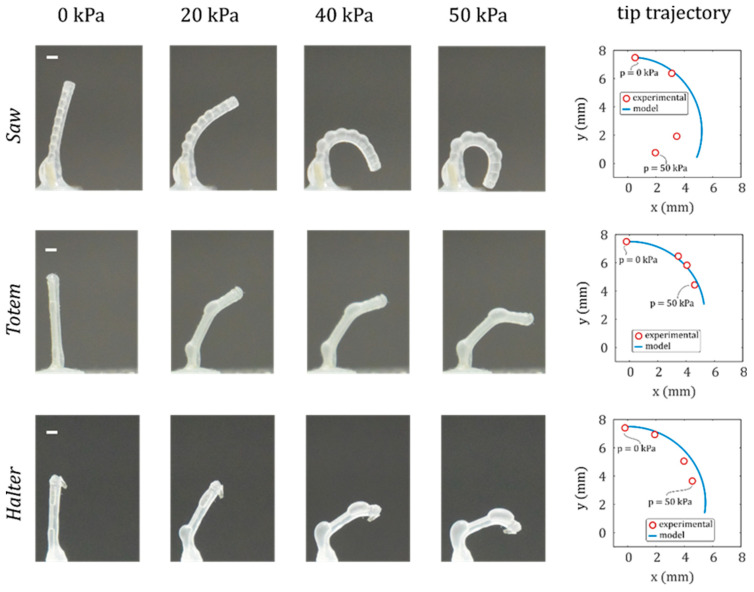
Microactuator inflation tests. Deformed configuration at four different pressures is reported for each microactuator. The white dash in the subfigures of the first column corresponds to a length of 0.8 mm. The experimental and modelled tip trajectory with respect to the initial position at 0 pressure is reported in the graphs.

The three microactuators are experimentally characterised using the setup described in Section 2.5. Figure 10 shows the deformation of the three microactuators at a pressure of 20, 40 and 50 kPa. The deformed shapes are in agreement with the results of the analytical model, showing the predicted segmented curvatures according to the shape of the inflatable cavity, as discussed in the previous paragraph.

However, the experimental displacement starts to deviate from the model at large displacements. This is more significant for actuators *Saw* and *Halter* as they have a higher distribution of thinner membranes that undergo large strains. Indeed, the assumptions of the linear model (such as linear elasticity and undeformed cross-sections) hold at small deformations, but for large displacements silicone rubbers follow hyperelastic models, where stress and strain are nonlinearly related, and cross sections deform. Moreover, circumferential strains become important at large deformations, leading to nonlinear phenomena that occur in rubber structures, such as ballooning and elastic instabilities [41]. This is one of the main reasons why soft bending actuators are manufactured with fibre-reinforcements or bellows shapes to limit circumferential strains [22,38]. Given the asymmetric geometry of the cross-section of our microactuators, the analytical formulation of the nonlinear problem is not trivial, and finite element method (FEM) is commonly used to deal with these nonlinearities [42].

Thanks to the segmented shape of the inner chamber introduced with the WEDG process, the ballooning effect is limited to the segments with lower stiffness and does not affect the whole actuator. For example, the 400 µm segments in *Saw* work as circumferential strain limiters and prevent the propagation of the ballooning from the 500 µm segments. This decreases the risk of bursting and allows the microactuator to safely achieve a fully curled configuration.

Another interesting feature that we obtained with WEDG can be observed in the *Totem* deformation (Figure 10, second row), where the low-stiffness segments balloon and bend while the rest of the cavity is less deformed, resulting in a finger-like motion. Therefore, larger bending deformations can be locally concentrated in the actuator.

## 4. Conclusions

In this paper we investigated a new production technology to improve the design of soft inflatable bending microactuators and achieving more complex deformations. WEDG processing was shown to accurately machine micromoulds whose shapes are replicated in the soft actuators through a bonding-free micromoulding process. As demonstrators, we proposed three different actuators that share the same global geometry and material except for the shape of the inflatable cavity. The actuators showed very different kinematics. To predict the response, we applied a simple analytical model based on a multi-segment approximation and linear beam theory. Experimental results agreed with the model-based predictions, within the limits posed by the linear approximation. We manufactured microactuators that exhibit application-relevant behaviours such as full curl, flexible joint-like fingers and undeformed segments. Indeed, this type of actuator has been used to develop flexible microgrippers [36], as well as biomedical devices [25]. In the future we envision a reverse kinematics approach in the form of an optimisation algorithm that, starting from a given trajectory of the end effector, is able to deduce the right morphology of the inflatable cavity which is compatible with WEDG manufacturing.

## Figures and Tables

**Table 1:** Inflatable cavities segments lengths.

Morphology	Segments	Lengths (mm)
*Saw*	20	0.4–0.35 (×10)
*Totem*	5	1.8–0.7–2.5–0.7–1.8
*Halter*	3	3–1.5–3

**Table 2:** Wire electrical discharge grinding (WEDG) processing parameters.

Parameter	Symbol	Unit	Machining Regime	
			Roughing	Finishing
Open voltage	*U*_0_	V	120	85
Capacitance ^1^	*C*	nF	5	1.5
Pulse-on time	*T_ON_*	μs	5	4
Pulse-off time	*T_OFF_*	μs	3	2
Reference voltage	*U_e_*	V	85	72
Spindle rotation	*R*	rev/min	850	700

^1^ Estimated value.

## References

[1] Rus D., Tolley M.T. (2015). Design, fabrication and control of soft robots. Nature.

[2] Kim S., Laschi C., Trimmer B. (2013). Soft robotics: A bioinspired evolution in robotics. Trends Biotechnol..

[3] Shintake J., Cacucciolo V., Floreano D., Shea H. (2018). Soft Robotic Grippers. Adv. Mater..

[4] Soft Robotics Inc. https://www.softroboticsinc.com.

[5] Hawkes E.W., Blumenschein L.H., Greer J.D., Okamura A.M. (2017). A soft robot that navigates its environment through growth. Sci. Robot..

[6] Shepherd R.F., Ilievski F., Choi W., Morin S.A., Stokes A.A., Mazzeo A.D., Chen X., Wang M., Whitesides G.M. (2011). Multigait soft robot. Proc. Natl. Acad. Sci. USA.

[7] Wehner M., Truby R.L., Fitzgerald D.J., Mosadegh B., Whitesides G.M., Lewis J.A., Wood R.J. (2016). An integrated design and fabrication strategy for entirely soft, autonomous robots. Nature.

[8] Hines L., Petersen K., Lum G.Z., Sitti M. (2017). Soft Actuators for Small-Scale Robotics. Adv. Mater..

[9] Konishi S. Small, soft, safe micromachine for minimally invasive surgery. Proceedings of the IMFEDK International Meeting for Future of Electron Devices.

[10] De Greef A., Lambert P., Delchambre A. (2009). Towards flexible medical instruments: Review of flexible fluidic actuators. Precis. Eng..

[11] Mosadegh B., Tavana H., Lesher-Perez S.C., Takayama S. (2011). High-density fabrication of normally closed microfluidic valves by patterned deactivation of oxidized polydimethylsiloxane. Lab Chip.

[12] Den Toonder J.M.J., Onck P.R. (2013). Microfluidic manipulation with artificial/bioinspired cilia. Trends Biotechnol..

[13] Gorissen B., Reynaerts D., Konishi S., Yoshida K., Kim J.-W., De Volder M. (2017). Elastic Inflatable Actuators for Soft Robotic Applications. Adv. Mater..

[14] Suzumori K., Iikura S., Tanaka H. Development of flexible microactuator and its applications to robotic mechanisms. Proceedings of the IEEE International Conference on Robotics and Automation.

[15] Wakimoto S., Ogura K., Suzumori K., Nishioka Y. Miniature soft hand with curling rubber pneumatic actuators. Proceedings of the IEEE International Conference on Robotics and Automation.

[16] Konishi S., Kawai F., Cusin P. (2001). Thin flexible end-effector using pneumatic balloon actuator. Sens. Actuators A Phys..

[17] Polygerinos P., Correll N., Morin S.A., Mosadegh B., Onal C.D., Petersen K., Cianchetti M., Tolley M.T., Shepherd R.F. (2017). Soft Robotics: Review of Fluid-Driven Intrinsically Soft Devices; Manufacturing, Sensing, Control, and Applications in Human-Robot Interaction. Adv. Eng. Mater..

[18] Gorissen B., Vincentie W., Al-Bender F., Reynaerts D., De Volder M. (2013). Modeling and bonding-free fabrication of flexible fluidic microactuators with a bending motion. J. Micromech. Microeng..

[19] Connolly F., Walsh C.J., Bertoldi K. (2016). Automatic design of fiber-reinforced soft actuators for trajectory matching. Proc. Natl. Acad. Sci. USA.

[20] Chou C.P., Hannaford B. (1996). Measurement and modeling of McKibben pneumatic artificial muscles. IEEE Trans. Robot. Autom..

[21] Overvelde J.T.B., Kloek T., D’haen J.J., Bertoldi K. (2015). Amplifying the response of soft actuators by harnessing snap-through instabilities. Proc. Natl. Acad. Sci. USA.

[22] Mosadegh B., Polygerinos P., Keplinger C., Wennstedt S., Shepherd R.F., Gupta U., Shim J., Bertoldi K., Walsh C.J., Whitesides G.M. (2014). Pneumatic networks for soft robotics that actuate rapidly. Adv. Funct. Mater..

[23] Gorissen B., De Volder M., De Greef A., Reynaerts D. (2011). Theoretical and experimental analysis of pneumatic balloon microactuators. Sens. Actuators A Phys..

[24] Milana E., Gorissen B., Peerlinck S., De Volder M., Reynaerts D. (2019). Artificial Soft Cilia with Asymmetric Beating Patterns for Biomimetic Low-Reynolds-Number Fluid Propulsion. Adv. Funct. Mater..

[25] Gorissen B., De Volder M., Reynaerts D. (2018). Chip-on-tip endoscope incorporating a soft robotic pneumatic bending microactuator. Biomed. Microdevices.

[26] Masuzawa T., Fujino M., Kobayashi K., Suzuki T., Kinoshita N. (1985). Wire Electro-Discharge Grinding for Micro-Machining. CIRP Ann. Manuf. Technol..

[27] Fleischer J., Masuzawa T., Schmidt J., Knoll M. (2004). New applications for micro-EDM. J. Mater. Process. Technol..

[28] Wang Y.-Q., Bellotti M., Li Z., Qian J., Reynaerts D. Twin-wire electrical discharge grinding for shaping tapered micro rods. Proceedings of the 19th International Conference and Exhibition of the European Society for Precision Engineering and Nanotechnology, EUSPEN 2019.

[29] Wang Y., Chen X., Gan W., Wang Z., Guo C. (2016). Complex Rotary Structures Machined by Micro-WEDM. Procedia CIRP.

[30] Sheu D.Y. (2010). Study on an evaluation method of micro CMM spherical stylus tips by µ-EDM on-machine measurement. J. Micromech. Microeng..

[31] Diver C., Atkinson J., Helml H.J., Li L. (2004). Micro-EDM drilling of tapered holes for industrial applications. J. Mater. Process. Technol..

[32] Zhang L., Tong H., Li Y. (2015). Precision machining of micro tool electrodes in micro EDM for drilling array micro holes. Precis. Eng..

[33] De Volder M., Peirs J., Reynaerts D., Coosemans J., Puers R., Smal O., Raucent B. (2005). Production and characterization of a hydraulic microactuator. J. Micromech. Microeng..

[34] Bellotti M., Milana E., Gorissen B., Qian J., Reynaerts D. Wire electrical discharge grinding of micro rods for bonding-free fabrication of soft pneumatic microactuators. Proceedings of the 18th International Conference and Exhibition of the European Society for Precision Engineering and Nanotechnology, EUSPEN 2018.

[35] Bellotti M., Qian J., Reynaerts D. (2018). Enhancement of the micro-EDM process for drilling through-holes. Procedia CIRP.

[36] Milana E., Bellotti M., Gorissen B., De Volder M., Reynaerts D. Precise bonding-free micromoulding of miniaturized elastic inflatable actuators. Proceedings of the RoboSoft 2019–2019 IEEE International Conference on Soft Robotics.

[37] Paek J., Cho I., Kim J. (2015). Microrobotic tentacles with spiral bending capability based on shape-engineered elastomeric microtubes. Sci. Rep..

[38] Polygerinos P., Wang Z., Overvelde J.T.B., Galloway K.C., Wood R.J., Bertoldi K., Walsh C.J. (2015). Modeling of Soft Fiber-Reinforced Bending Actuators. IEEE Trans. Robot..

[39] Jahan M.P., Rahman M., Wong Y.S. (2011). A review on the conventional and micro-electrodischarge machining of tungsten carbide. Int. J. Mach. Tools Manuf..

[40] Delaney K.D., Bissacco G., Kennedy D. Demoulding force prediction for micro polymer replication: A review of relevant literature. Proceedings of the 6th International Conference on Multi Material Micro Manufacture (4M).

[41] Gent A.N. (2005). Elastic instabilities in rubber. Int. J. Non-Linear Mech..

[42] Moseley P., Florez J.M., Sonar H.A., Agarwal G., Curtin W., Paik J. (2016). Modeling, Design, and Development of Soft Pneumatic Actuators with Finite Element Method. Adv. Eng. Mater..

